# Investigating physical fitness and race performance as determinants for the ACL injury risk in Alpine ski racing

**DOI:** 10.1186/s13102-016-0049-6

**Published:** 2016-08-17

**Authors:** Kai-Uwe Schmitt, Nicole Hörterer, Michael Vogt, Walter O. Frey, Silvio Lorenzetti

**Affiliations:** 1 Institute for Biomedical Engineering, University and ETH Zurich, AGU Zurich (Working Group on Accident Mechanics), Winkelriedstrasse 27, 8006 Zurich, Switzerland; 2 Institute for Biomechanics, ETH Zurich, Leopold-Ruzicka-Weg 4, 8093 Zurich, Switzerland; 3 Swiss Ski Federation, Haus des Skisports, Worbstrasse 52, Postfach 252 3074, Muri bei, Bern Switzerland; 4 Balgrist MoveMed, Swiss Olympic Medical Center, University Hospital Balgrist, 8008 Zurich, Switzerland

**Keywords:** Athletic injuries, Anterior cruciate ligament (ACL), Knee injury, Skiing, Risk

## Abstract

**Background:**

ACL ruptures in Alpine ski racers are frequently observed. This study analysed the association between physical fitness, race performance and the knee injury history.

**Methods:**

A retrospective study was conducted to investigate the influence of physical fitness and performance on the knee injury outcome. As part of this study an injury data base (covering 2004–2013) was established that recorded information about the athletes, their fitness status as determined by a standardised fitness test (Swiss Ski Power Test, SSPT) as well as medical information related to injuries. The performance of athletes who sustained knee injury was compared to athletes who suffered no injury or a different injury.

**Results:**

Twenty-seven (19f, 8 m) of 70 athletes sustained a knee injury. ACL ruptures accounted for 71 % of these knee injuries. While more females sustained a knee injury, the difference between males and females was not statistically significant. It was shown that athletes with a better FIS (Fédération Internationale de Ski) rank were more prone to knee injury. However, none of the parameters related to physical fitness was linked to a history of knee injury.

**Conclusions:**

A general fitness test as SSPT is not associated with a history of knee injury in Alpine skiing. More specific physical fitness test procedures should be investigated to determine relevant fitness factors.

## Background

Knee injuries are a major concern in Alpine ski racing whereas ruptures of the anterior cruciate ligament (ACL) are most frequently observed [1–4]. However, analysing injuries sustained during WorldCup races, sex differences in the rate of ACL injuries were not observed [5]. The consequences of such injury can be severe and might include the need for extensive medical treatment and rehabilitation, possible long-term effects such as a reduced stability of the knee or even the end of the athlete’s career [4, 6–8]. Thus there is an interest in developing measures to reduce the injury risk. This requires the identification of corresponding injury risk factors. Different risk factors were analysed with respect to traumatic injuries in skiing and particularly regarding ACL ruptures including physical fitness as one potential risk factor [4].

Physical fitness as composed of strength, endurance, coordination and flexibility is discussed to be related to injury risk in different sports. However, controversial results were presented. Most recently Grant et al. [9] reported that aerobic fitness and maximum strength outcomes were not strongly predictive for injury in ice hockey. When investigating whether pre-season fitness measures can predict time to injury in varsity athletes Kennedy et al., [10] found that time to injury was influenced most heavily by gender and sport, but not by fitness measures. McGill et al. [11] analysed the predicting performance and injury resilience from movement quality and fitness scores in a basketball team, but no patterns with injury emerged. Looking at the association between pre-season functional tests and injuries in youth football, only physical fatigue was significantly linked to injury [12] In contrast, improvements in aerobic-running fitness and increased body mass seem to protect against in-season injury in elite football [12]. In a study by Chalmers et al. [13] also lower aerobic endurance, faster 5-m acceleration and greater planned agility were associated with an increased risk of various injury types in elite junior football players. With respect to skiing, Raschner et al. [4] evaluated a battery of 9 different tests and reported that low strength in the upper body and the legs were associated with a high knee injury risk in skiing.

In view of the fact that sports federations regularly test the physical fitness of their athletes, it would be very valuable to be able to identify athletes with a higher risk of knee injury based on fitness data. In 2004, Swiss Ski, the national skiing federation, introduced a new test battery called Swiss Ski Power Test (SSPT) to assess the physical fitness of their athletes. The test is composed of eight different exercises and it is meant to be a general fitness test used to monitor the fitness level of the athletes. Since ACL ruptures are also of major concern in the Swiss Alpine ski racing teams, the aim of this study was to investigate retrospectively whether physical fitness is associated with knee injury history, especially ACL rupture. It was hypothesised that the performance of an athlete in the SSPT would be lower in those with a history of ACL injury, i.e., it was tested whether any of the components of the SSPT correlates with the actual injury outcome of the athletes. In addition, it was investigated whether the performance during a standardised muscle performance test including isometric contractions, and counter movement and squat jumps is associated with a history of ACL injury, i.e., it was tested whether a muscle performance test is linked to injury risk.

## Methods

As part of this study an injury data base was established [14]. In line with Swiss legislation (Human Research Act) athletes who agreed to participate in this retrospective study gave written consent that their data, particularly medical information, could be collected and used in anonymous form. The entire procedure of this study including the collection and the analysis of the data, was audited by the local ETH ethics committee.

### Data sampling

Athletes of Swiss Ski who were a member of a higher level national team (i.e., National Team, A, B or C squad) and who trained at one of the national elite sports centres during the period of 2004–2013 were invited to participate in this study by means of an online questionnaire. This included athletes of all Alpine disciplines (downhill skiing, super giant slalom (super G), giant slalom, slalom as well as super combination). For each athlete the fitness data as recorded during his or her career was included in the data base. Furthermore, their performance was represented by their corresponding FIS scores (FIS: Fédération Internationale de Ski). Here a low score represents a better overall ranking.

Those athletes who claimed that they had sustained any type of acute injury on the musculoskeletal system were asked, within the online questionnaire, for permission to use their medical data. Seeking medical assistance was used as definition of injury. Only a diagnosis and medical information that was confirmed by the athlete’s clinician (medical physician) was included in the data base.

### Fitness and performance data

The Swiss Ski Power Test (SSPT) is a test procedure to establish an overall status of the physical fitness [15]. The SSPT was introduced in 2004 and is nowadays established as the standard test for performance and talent selection [16]. The test is composed of eight different tasks to test aerobic fitness, anaerobic fitness, strength of the legs and the trunk, coordination and speed (Table 1). All athletes performed the tests in a standardised way whereas athletes conduct the SSPT at the start of their career and various times during their career. However, the number of tests during their career does not follow a strict procedure; the official guideline by Swiss Ski suggests performing the test twice per year. In the period of 2004 to 2013 a total of 424 national level athletes (192 males, 232 females) have performed 2503 SSPTs. Each athlete performed the SSPT several times a year from the beginning to the end of his/her career whereas the tests were performed decentralized following a standardized and instructed procedure [15]. This data was used to generate an age-specific reference data set which describes the average performance of the athletes.

**Table 1:** The different tasks of the Swiss Ski Power Test (SSPT) [15]

Task	Test parameter (s)	Measure
Swiss Cross	speed/coordination	time [s]
push-ups	strength endurance upper body	time [s]
one leg 5-hop	speed/strength	distance [m]
standing long jump	speed/strength	distance [m]
plank test	force endurance trunk	time [s]
obstacle run	coordination	time [s]
high box jump	anaerobic endurance	repetitions within 90 s
12 min run	aerobic endurance	distance [m]

Note: The plank test was replaced in 2013 by the trunk twist test, i.e., for the plank test which was considered in this study only data from 2004 to 2012 was available

In addition to the Swiss Ski Power test, available data of the MLD [17] test with three different isometric and dynamic disciplines was included. The MLD test analysis the maximum isometric leg strength of the extensor muscles (MVC, maximal force, N/kg, normalized to BW) in a squat position at a knee angle of 100° with a fixed bar at shoulder level. Furthermore, the maximal jump performance of counter movement jumps (CMJ, power per BW, W/kg) and squat jumps (SJ, power per BW, W/kg) were recorded bipedal. Also for this data a reference was established: average values (specific for sex and squad level) were calculated based on data from all athletes who were tested in the period of 2000 to 2013. MLD data of those who sustained a knee injury was assessed by dividing the value available in the year of injury by the average value of the corresponding reference sample of this year. Thus the performance of the athlete relative to the corresponding age-match average was assessed. For this assessment a corridor of 3 % was set, i.e., the performance was regarded as better than average if the ratio was above 1.03, worse if the ratio was below 0.97 and average if the ratio ranged between 0.97 and 1.03.

The FIS score of an athlete at the end of a season was also added to the data base. For each discipline the score is established according to the rules of the FIS. The FIS system is a penalty system such that a lower score indicates a better performance. To ensure that only data of actively participating athletes was evaluated, it was decided to exclude all scores of 150 or higher from this analysis as athletes with such high scores do apparently not race often. In this study the performance of injured athletes was characterised by their FIS score at the time of injury. Regarding SSPT and the MLD test, the most recent data available before injury was evaluated (up to 1 year before injury). Furthermore, the FIS rank was considered. The FIS rank represents the official ranking based on the FIS scores.

### Statistical evaluation

All statistical analyses were performed using the software IBM SPSS Statistics (version 22.0.0). Descriptive statistics was used to characterise the data sample. The data was tested for normal distribution using Q-Q-plots and Kolmogorov–Smirnov test. Ordinal data is presented using average and standard deviation while nominal data is given in percentages. A binomial test (corrected for different sample sizes) was used to test for the influence of sex on knee injury prevalence. For further analysis the sample was divided into three groups: athletes without injuries, athletes with knee injury and athletes with any other injury. The first two groups were in the focus of the study presented here. Differences between these groups were analysed using single factor analysis of variance (ANOVA) for each test and discipline; significant results were checked using Scheffé post-hoc test or Tamhane-T2, respectively, depending on homogeneity of variance. α was set to 0.05 in all tests.

## Results

Seventy athletes agreed to participate in this study and their data was recorded in the injury data base (Table 2). Twenty athletes did not sustain an injury, 27 suffered from a knee injury and 23 persons sustained a different injury (such as head injury or injuries to the upper extremities) during the period of 2004 to 2013. Tables 3, 4 and 5 show the FIS scores, the results of the SSPT and the MLD test, respectively.

**Table 2:** Population evaluated in this study

Group	Number of athletes [n]	Age [years ± SD]	Height [cm ± SD]	Weight [kg ± SD]	BMI [kg/m^2^ ± SD]	SSPT [n]	FIS [n]
All							
no injury	20	14.7 ± 1.9	163 ± 12	58 ± 15	21.6 ± 3.1	141	81
knee injury	27	17.5 ± 2.2	167 ± 6	63 ± 7	22.4 ± 1.8	15	26
other injury	23	15.1 ± 2.1	166 ± 11	61 ± 15	21.6 ± 2.9	151	112
total	70	15.1 ± 2.1	165 ± 11	60 ± 14	21.6 ± 3.0	307	219
Males							
no injury	10	15.1 ± 1.9	168 ± 11	65 ± 13	22.6 ± 2.5	81	45
knee injury	8	17.7 ± 2.3	172 ± 3	66 ± 7	22.2 ± 1.8	3	7
other injury	14	15.0 ± 2.0	169 ± 12	50 ± 12	21.9 ± 3.5	87	68
total	32	15.0 ± 2.0	169 ± 11	64 ± 15	22.3 ± 3.0	171	120
Females							
no injury	10	14.3 ± 1.9	156 ± 9	50 ± 12	20.1 ± 3.2	60	36
knee injury	19	17.4 ± 2.2	166 ± 6	62 ± 7	22.5 ± 1.9	12	19
other injury	9	15.4 ± 2.2	163 ± 7	56 ± 8	21.1 ± 1.9	64	44
total	38	15.1 ± 2.2	160 ± 8	55 ± 10	20.8 ± 2.6	136	99

SSPT and FIS denote for the number of Swiss Ski Power Tests and FIS scores, respectively, that were available

**Table 3:** Summary of the FIS scores achieved in different disciplines

	FIS Scores				
Group	Downhill	Slalom	Giant slalom	Super G	Super combination
no injury	76 ± 38	58 ± 23	42 ± 22	62 ± 33	72 ± 42
knee injury	54 ± 39	43 ± 32	30 ± 14	40 ± 30	59 ± 42
other injury	77 ± 40	55 ± 25	53 ± 26	67 ± 32	79 ± 40
total	73 ± 39	55 ± 26	46 ± 24	62 ± 34	73 ± 41

For each athlete of our study group all available scores were evaluated

**Table 4:** Results of the Swiss Ski Power Test per task for all athletes of our study group

	SSPT								
Group	Swiss Cross [s]	Push-ups [s]	One leg 5-hop left [m]	One leg 5-hop right [m]	Standing long jump [m]	Plank test [s]	Obstacle run [s]	High box jump 90 [#]	12 min run [m]
no injury	13.8 ± 1.0	88.4 ± 23.1	10.5 ± 1.5	10.3 ± 1.5	2.2 ± 0.3	230 ± 89	24.9 ± 3.0	76 ± 15	2709 ± 321
knee injury	13.5 ± 0.8	89.9 ± 26.4	10.3 ± 1.1	10.6 ± 1.2	2.2 ± 0.2	257 ± 101	24.4 ± 2.6	84 ± 10	2768 ± 227
other injury	13.6 ± 1.0	82.2 ± 27.4	10.8 ± 1.5	10.5 ± 1.4	2.2 ± 0.3	258 ± 101	24.5 ± 2.7	78 ± 15	2741 ± 299
total	13.7 ± 1.0	87.6 ± 25	10.7 ± 1.4	10.4 ± 1.4	2.2 ± 0.3	245 ± 96	24.7 ± 2.9	77 ± 15	2728 ± 306

**Table 5:** Performance of the reference group (uninjured skiers) in the different tasks of the MLD test

	MLD Reference Group											
Squad	MVC [N/kg]				CMJ [W/kg]				SJ [W/kg]			
	Male	[n]	Female	[n]	Male	[n]	Female	[n]	Male	[n]	Female	[n]
C	39.37	101	35.05	56	57.78	147	48.58	56	56.37	147	46.45	56
B	39.26	83	36.16	78	58.90	125	47.78	78	56.90	125	45.58	78
A	39.13	46	39.16	95	59.38	77	48.73	95	57.18	77	46.11	95

This data was used to compare the performance of our study group with respect to the average (matched for squad and sex)

### Knee injuries

Nineteen females and eight males reported knee injury. Although the number of females is clearly larger, the difference is statistically not significant (*p* = 0.067). Those 27 athletes reported a total of 38 knee injuries. Seventy-one percent of the injuries involved the ACL: 10 injuries were isolated ACL ruptures, 17 injuries were ACL ruptures in combination with a lesion of the lateral ligaments and/or the menisci and 11 injuries did not affect the ACL, but other knee structures such as the menisci.

### Risk factors analysis

**Fig. 1:**
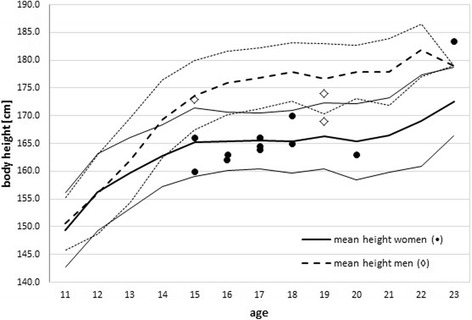
Body height and age of all individuals of the knee injury group (women: dots; men: diamonds) compared to the reference group of all Swiss Ski athletes. Although most athletes with knee injury seem smaller than average, the difference is not statistically significant (lines, average ± SD)

Table 6 summarises the results of the statistical analysis checking for differences of the three study groups with respect to body weight, height, body-mass-index (BMI), the result of the fitness test and the FIS score. Statistically significant differences were only found for body height and the FIS scores in some disciplines. The group with other injuries (i.e., no knee injury) was taller than the group without injuries, but there was no difference related to the group with knee injury. In addition, Fig. 1 illustrates the distribution of age and body height of the all the individuals of the knee injury group compared to the corresponding age-matched reference group.

**Table 6:** Analysis of differences among the three groups

Parameter	ANOVA, *p*-value	Post hoc test
weight [kg]	ns	
height [cm]	0.018*	*p* = 0.075, Tamhane-T2, knee injury vs. no injury *p* = 0.948, Tamhane-T2, knee injury vs. other injury *p* = 0.025*, Tamhane-T2, no injury vs. other injury
BMI	ns	
SSPT	ns	
FIS score (downhill)	0.039*	*p* = 0.067, Scheffé, knee injury vs. no injury *p* = 0.051, Scheffé, knee injury vs. other injury *p* = 0.989, Scheffé, no injury vs. other injury
FIS score (slalom)	ns	
FIS score (giant slalom)	0.000*	*p* = 0.062, Scheffé, knee injury vs. no injury *p* = 0.000*, Scheffé, knee injury vs. other injury *p* = 0.008*, Scheffé, no injury vs. other injury
FIS score (super G)	0.001*	*p* = 0.009*, Scheffé, knee injury vs. no injury *p* = 0.001*, Scheffé, knee injury vs. other injury *p* = 0.686, Scheffé, no injury vs. other injury
FIS score (super combination)	ns	

*ns* not significant

*p ≤ 0.05

Athletes participating in the discipline of slalom sustained ACL ruptures most often. Those who sustained a knee injury had a better FIS score compared to the other two study groups in the disciplines downhill, giant slalom and super G slalom. Analysing the FIS ranks (instead of the FIS scores) showed that athletes who sustained knee injuries were ranked better in nearly all disciplines (Table 7).

**Table 7:** Results of post-hoc tests related to the FIS ranks (which are based on FIS scores) show that athletes with knee injury were generally ranked better

Group	Downhill	Slalom	Giant slalom	Super G	Super combination
knee injury vs. no injury	0.005*^a^	0.013*^b^	0.001*^a^	0.001*^a^	0.063
knee injury vs. other injury	0.001*^a^	0.017*^b^	0.001*^a^	0.001*^a^	0.002*^a^

*p ≤ 0.05

^a^Tamhane T2, ^b^Scheffé

**Fig. 2:**
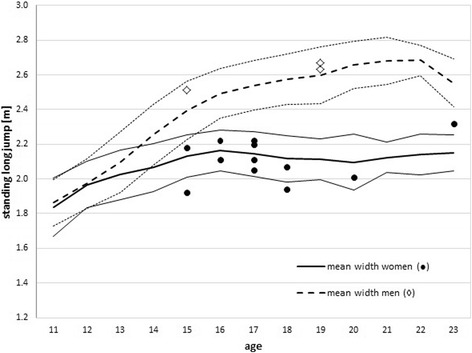
The performance of all individuals of the knee injury group (women: dots; men: diamonds) in the standing long jump task that is part of the SSPT compared to the reference group of all athletes (lines, average ± SD)

The fitness test did not account for any statistically significant difference between the three groups. This holds true for all components of the SSPT; when checking the results of the tasks that make up the SSPT no difference between the three groups was found. Figure 2 presents a typical example of one component of the SSPT.

In addition to the SSPT, MLD test results were available for 17 of the 27 athletes in the knee injury group (Table 8). For maximum voluntary contraction (MVC) 10 of the 17 athletes performed worse than the average performance of the corresponding reference group. For counter movement jumps (CMJ), in contrast, the athletes with knee injuries had a record of results above average (12 of 17 athletes performed better than average). In squat jumps (SJ) the performance of the knee injury group was average or above (7 of 17 performed average and also 7 of 17 performed above average).

**Table 8:** Results of the tests checking for leg strength (MLD test)

Subject No.	Sex	Squad	MVC [N/kg]	Performance relative to average + better 0 equal - poorer	CMJ [watt/kg]	Performance relative to average + better 0 equal - poorer	SJ [watt/kg]	Performance relative to average + better 0 equal - poorer
1	m	C	35.8	-	65.0	+	57.4	0
2	m	A	49.9	+	57.1	-	55.5	0
3	m	B	30.9	-	54.1	-	51.2	-
4	m	B	44.4	+	64.7	+	58.9	+
5	m	A	37.0	-	71.0	+	59.2	+
6	m	B	42.7	+	67.1	+	57.8	0
7	w	C	33.5	-	47.4	0	47.7	0
8	w	A	29.2	-	49.3	0	49.7	+
9	w	B	35.6	0	50.8	+	47.0	+
10	w	C	29.5	-	51.5	+	47.5	0
11	w	C	34.9	0	47.2	0	43.8	-
12	w	A	31.5	-	54.1	+	42.5	-
13	w	C	33.5	-	50.9	+	45.5	0
14	w	C	32.5	-	53.3	+	49.3	+
15	w	A	Na		62.6	+	53.7	+
16	w	C	36.3	+	54.7	+	46.2	0
17	w	C	29.7	-	54.5	+	51.4	+

The results of each athlete of the knee injury group were compared to the average value of a reference group of same sex, age and squad level. A performance of more than 103 % was regarded better than average, a performance below 97 % was regarded poorer

## Discussion and implications

Data on physical fitness, performance and injuries was collected and evaluated in this retrospective study. A corresponding injury data base was established and served as the basis for this analysis. The establishment of such a data base is regarded as an important step towards systematic monitoring of injuries sustained in ski racers. The data base allows linking injury data to fitness data as well as other performance data of the athletes.

### Knee injury risk

The difference of incidence of ACL injuries between injured male and female athletes was statistically not significant. It might be speculated that with a higher number of participants, it would have been possible show that females suffered more such injury than males.

This finding is in line other studies on injuries in competitive skiers [1, 18]. Also the average age of the ski racers who suffered a knee injury in our study was in the range (total 21 ± 3.8 years) that is reported in other work [18]. Furthermore, our study confirmed that ACL ruptures are the most frequent knee injury in competitive alpine skiing [1, 2]. Thus we conclude that our sample can be regarded as representative with regard to Alpine ski racing; additionally the three different groups that were analysed here included similar numbers of persons which allowed for valid statistical evaluation.

Athletes participating in the discipline of slalom seem to sustain ACL ruptures most often. This finding might be attributed to the fact that young athletes at the beginning of their career participate more often in slalom or giant slalom. Participation in disciplines such as downhill and super G slalom are associated with higher speeds which is a factor thought to be responsible for a higher injury risk by some [1, 7]. This might be more common for more experienced racers and could thus be underrepresented in our sample.

In contrast to studies on recreational skiers [2] or military personnel [19] neither body weight nor BMI could be linked to the knee injury risk. Also the body height of the knee injury group was not statistically related to the injury outcome (Fig. 1). Furthermore, it should be noted that most athletes were competing during adolescence. Here an enhancement of the performance could be influenced by training but also due to maturing.

### Physical fitness

The statistical analysis showed that a general fitness test like the Swiss Ski Power Test (SSPT) is not associated with ACL injury in Alpine skiing racers, i.e., it is not suitable predictor for such injury. This finding is in line with a recently published review by Bahr et al. [20]. None of the tasks that are included in the SSPT battery and which test for different aspects of physical fitness could be linked statistically to the ACL injury group. This result is to some extent surprising as good leg and trunk strength were often suspected to be determinants for the knee injury risk [4, 19, 21] and the SSPT includes several tasks in this area. However, this result is generally in agreement with work by Raschner et al. [4] who have analysed the physical fitness of skiers based on different parameters than those included in the SSPT. Also in their work most fitness parameters did not relate to the knee injury risk; only trunk strength was linked to the ACL rupture risk. A similar tendency seems also to be included in our data. As indicated in Fig. 2, the knee injury group performed average or poorer in the standing long jump when compared to the reference group of the same age and they showed a below average MVC (maximum voluntary contraction) performance. To achieve a good MVC performance requires good strength of legs, but also the ability to stabilise the trunk. Hence a poor performance in the MVC test might be related to poor strength of the trunk and thus influence the ACL injury risk as described by Raschner et al. [4]. Our finding that the injured athletes performed poorly in the MVC test, but quite well in counter movement jumps (CMJ) and squad jumps (SJ) could be explained by the fact that the MVC does primarily depend on the muscle cross-sectional area and less on the fibre type composition of the muscle (which is relevant for the CMJ and SJ performance). Also well-coordinated neuronal activation can influence the outcome of the leg strength test.

In conclusion it can be stated that the results of this study do not support a direct correlation of physical fitness and the ACL injury risk, but there are indications that some factors such as the (isometric) leg strength might nonetheless influence the ACL injury risk as suggested by other work [4]. Bearing in mind that the SSPT covers a wide range of tasks, a more specific fitness test might, however, be able to establish a better link to the injury risk. Further research in this area is needed and the continuous use of data bases to document injury as well as fitness parameters is recommended to systematically record relevant data.

In our data the FIS rank was related to the injury outcome. Partly also the FIS score was shown to correlate with the injury history, but the final FIS rank was statistically more clearly linked to ACL injury. Similarly Pujol et al. [18] found a higher prevalence of ACL ruptures in the top 30 world cup skiers. These findings are somewhat difficult to explain, but troublesome at the same time. Athletes with better FIS score (generally regarded as successful athletes) apparently face a higher injury risk, which poses a high risk to their career and which makes more efforts to protect the best athletes more demanding. However, this also suggests that athletes with better FIS score are not necessarily those who exhibit above average physical fitness based on the parameters used in this study. Therefore, either the currently used tests to determine individual fitness parameters are less suitable and/or there are other parameters that have a more significant impact on the injury risks. Future research is thus needed to also account for factors that might, for instance, be related to the risk taking behaviour or mental aspects of successful athletes – parameters which are currently not included in the standard records of the skiing association, but which might influence the ACL injury risk.

### Limitations

This study focussed on athletes of Swiss Ski. Whereas the selection criteria and the different test procedures are standardised within Swiss Ski, they surely differ from procedures of other national skiing associations. This represents a bias, which makes it more difficult to compare the results with published research based on other samples. Only including self-reported injury information that was confirmed by the treating clinician increased the quality of the medical data, but reduced the sample size. Due to the organisation of the decentralized testing of the SSPT, it might be possible that not all the athletes have the very same number of tests. This might influence the test performance. A further difficulty is related to the age match of the injury group and the reference group. Whereas the age of an athlete at the time of injury is clearly defined, the control group represents an age average. More details related to the specific injury mechanism, exposure data, some extrinsic factors related to the injurious event (e.g., weather conditions, equipment) or individual measures of the juvenile athletes such as anthropometric data or biological age could not be retrieved. This limits the use of more elaborated statistical methods.

## Conclusions

The retrospective analysis performed in this study did not reveal a link between the physical fitness of an athlete and the incidence of ACL injury. Consequently a general fitness test like the Swiss Ski Power Test (SSPT) seems not to be a good indicator for ACL injury in Alpine skiing racers. More specific fitness tests might be necessary to be able to establish a correlation between fitness and the risk of knee injury or ACL rupture, respectively. However, generally it remains questionable to what extend fitness tests can be related to injury prediction with sufficient accuracy [20].

However, the statistical analysis also showed that athletes who sustained ACL injury are associated with high FIS ranks, i.e., the risk of ACL injury is higher in athletes who had higher FIS scores, suggesting that those with better skiing-specific performance/rank had a higher incidence of knee injury. On the one hand this highlights the need for further research to reduce the risk of career threatening injuries in successful athletes. On the other hand this finding might also suggest that procedures to identify athletes who are at high risk of sustaining ACL injury might be more effective if they also consider other measures such as psychological factors, risk taking behaviour and neuromuscular aspects to characterise the athlete.

## Abbreviations

ACL: Anterior Cruciate Ligament
ANOVA: Analysis Of Variance
BMI: Body Mass Index
CMJ: Counter Movement Jump
FIS: Fédération Internationale de Ski
MLD: Muscle Performance Test
MVC: Maximum Voluntary Contraction
SJ: Squat Jump
SSPT: Swiss Ski Power Test
super G: Super Giant Slalom

## References

[1] Flørenes TW (2009). Injuries among male and female World Cup alpine skiers. Br J Sports Med.

[2] Ruedl G (2011). ACL injury mechanisms and related factors in male and female carving skiers: a retrospective study. Int J Sports Med.

[3] Bere T (2011). Mechanisms of anterior cruciate ligament injury in World Cup alpine skiing: a systematic video analysis of 20 cases. Am J Sports Med.

[4] Raschner C (2012). The relationship between ACL injuries and physical fitness in young competitive ski racers: a 10-year longitudinal study. Br J Sports Med.

[5] Bere T (2014). Sex differences in the risk of injury in World Cup alpine skiers: a 6-year cohort study. Br J Sports Med.

[6] Oetiker, R.F. Orthopädische Chirurgie Dr.med.Rolf F. Oetiker. 2009 July 10th 2009 [cited 2015 June 17th 2015]; Available from: http://www.orthozentrum.ch/Huefte-und-Knie/Kreuzbandriss.aspx. Accessed June 2015.

[7] Sporri J (2012). Perceived key injury risk factors in World Cup alpine ski racing--an explorative qualitative study with expert stakeholders. Br J Sports Med.

[8] Sadoghi P, von Keudell A, Vavken P (2012). Effectiveness of anterior cruciate ligament injury prevention training programs. J Bone Joint Surg Am.

[9] Grant JA (2015). Ability of preseason body composition and physical fitness to predict the risk of injury in male collegiate hockey players. Sports Health.

[10] Kennedy MD (2012). Can pre-season fitness measures predict time to injury in varsity athletes?: a retrospective case control study. Sports Med Arthrosc Rehabil Ther Technol.

[11] McGill SM, Andersen JT, Horne AD (2012). Predicting performance and injury resilience from movement quality and fitness scores in a basketball team over 2 years. J Strength Cond Res.

[12] Gastin PB (2015). Increase in injury risk with low body mass and aerobic-running fitness in elite Australian football. Int J Sports Physiol Perform.

[13] Chalmers DJ, Samaranayaka A, McNoe BM (2013). Risk factors for injury in community-level football: a cohort study. Int J Inj Contr Saf Promot.

[14] Kallen R (2014). Systematische Erfassung von Knieverletzungen im Schweizer Ski-Rennsport. Department of Health Sciences and Technology.

[15] Gorski T (2014). An anthropometric and physical profile of young Swiss alpine skiers between 2004 and 2011. Int J Sports Physiol Perform.

[16] Vogt, M., ed. Wissenschaft trifft Praxis: am Beispiel des Swiss-Ski Power Tests. 25 ed. Sport ist Spitze, ed. M.a.V. Bracht, L. 2010, Meyer & Meyer Aachen Germany. 47–50.

[17] Hübner K (2009). Veränderungen der Explosivkraft der unteren Extremitäten in Abhängigkeit vom Widerstand - Studie bei Schweizer Spitzensportlern aus Sportarten mit hohem Explosivkraftanteil.

[18] Pujol N, Blanchi MP, Chambat P (2007). The incidence of anterior cruciate ligament injuries among competitive alpine skiers. Am J Sports Med.

[19] LaBella CR (2014). Anterior cruciate ligament injuries: diagnosis, treatment, and prevention. Pediatrics.

[20] Bahr R (2016). Why screening tests to predict injury do not work—and probably never will…: a critical review. Br J Sports Med.

[21] Alentorn-Geli E (2009). Prevention of non-contact anterior cruciate ligament injuries in soccer players. Part 1: Mechanisms of injuries and underlying risk factors. Knee Surg Sports Traumatol Arthrosc.

